# The Role of Polymer Structure in Formation of Various Nano- and Microstructural Materials: 30 Years of Research in the Laboratory of Nano- and Microstructural Materials at the Centre of Polymer and Carbon Materials PAS †

**DOI:** 10.3390/polym13172892

**Published:** 2021-08-27

**Authors:** Natalia Oleszko-Torbus, Barbara Mendrek, Agnieszka Kowalczuk, Wojciech Wałach, Barbara Trzebicka, Alicja Utrata-Wesołek

**Affiliations:** Centre of Polymer and Carbon Materials, Polish Academy of Sciences, 41-819 Zabrze, Poland; noleszko@cmpw-pan.edu.pl (N.O.-T.); bmendrek@cmpw-pan.edu.pl (B.M.); akowalczuk@cmpw-pan.edu.pl (A.K.); wwalach@cmpw-pan.edu.pl (W.W.)

**Keywords:** polyoxiranes, poly(meth)acrylates, polyoxazolines, polymer architecture, branched polymers, block copolymers, star polymers, polymer nanostructure, polymer self-organization in solution, polymer nanolayers

## Abstract

The review summarizes the research carried out in the Laboratory of Nano- and Microstructural Materials at the Centre of Polymer and Carbon Materials, Polish Academy of Sciences (CMPW PAS). Studies carried out for many years under the guidance of Professor Andrzej Dworak led to the development and exploration of the mechanisms of oxirane and cyclic imine polymerization and controlled radical polymerization of methacrylate monomers. Based on that knowledge, within the last three decades, macromolecules with the desired composition, molar mass and topology were obtained and investigated. The ability to control the structure of the synthesized polymers turned out to be important, as it provided a way to tailor the physiochemical properties of the materials to their specific uses. Many linear polymers and copolymers as well as macromolecules with branched, star, dendritic and hyperbranched architectures were synthesized. Thanks to the applied controlled polymerization techniques, it was possible to obtain hydrophilic, hydrophobic, amphiphilic and stimulus-sensitive polymers. These tailor-made polymers with controlled properties were used for the construction of various types of materials, primarily on the micro- and nanoscales, with a wide range of possible applications, mainly in biomedicine. The diverse topology of polymers, and thus their properties, made it possible to obtain various types of polymeric nanostructures and use them as nanocarriers by encapsulation of biologically active substances. Additionally, polymer layers were obtained with features useful in medicine, particularly regenerative medicine and tissue engineering.

## 1. Introduction

The present state of knowledge and current investigations of macromolecules with varied topology carried out in the Laboratory of Nano- and Microstructural Materials are the consequence of the early research focused on basic relations in the polymerization processes of cyclic monomers, which began in the 1990s. The initial basic research on polymerization of cyclic monomers was started by Prof. Andrzej Dworak and his team at the Institute of Coal Chemistry of the Polish Academy of Sciences in Gliwice. The main directions of research conducted at that time were the cationic polymerization of oxiranes, particularly the glycidol monomer and 2-substituted-2-oxazolines. Early investigations in the case of glycidol, a monomer containing a hydroxyl group, were focused on the role of this group in the mechanism of its cationic polymerization. The study of the cationic polymerization of glycidol and the detailed analysis of the chain structure of the obtained hyperbranched polymers allowed us to determine the contribution of the active chain end (ACE) and activated monomer (AM) mechanisms in this process and made it possible, to some extent, to control the degree of branching of the resulting macromolecules. This work was performed in collaboration with the Centre of Molecular and Macromolecular Studies of the Polish Academy of Sciences in Łódź (CMMS PAS), where the activated monomer mechanism was previously discovered and explained in detail by Kubisa [1,2]. Later studies on the synthesis of linear glycidol polymers via controlled anionic polymerization of glycidol with a protected hydroxyl group under homogeneous conditions were the beginning of the development of methods for the synthesis of linear functional polymers and block copolymers based upon this monomer. The findings have enabled a number of syntheses of linear polymers, copolymers, macromonomers and more complex polymer structures, which are also thermoresponsive, to be developed and carried out.

At the same time, research was also being conducted on the mechanism of cationic polymerization of 2-oxazolines, which led to the discovery of the participation of ionic and covalent growth centers in this process. Research was started in Mainz in collaboration with Prof. R. C. Schulz [3,4,5]. The use of new mono- and multifunctional initiators resulted in the synthesis of many linear and branched polymers and copolymers of 2-substituted-2-oxazolines.

Independent of synthetic research, the laboratory gained experience in polymer analysis and characterization. A research apparatus based on static and dynamic light scattering, with which the laboratory is equipped and which is necessary for the determination of molar masses of polymers and nanoparticles, allowed for precise characterization of even complex polymer structures and for studies on polymer organization in solution. Based on this knowledge, research in the laboratory has been directed toward more complex processes of macromolecule organization in solutions, particularly for thermoresponsive polymers. The ability to very precisely characterize the obtained polymers has enabled the synthesis of macromolecules with branched structures, such as grafted polymers, bottle brush polymers, stars, dendritic polymers, core–shell polymers and others. The laboratory cooperated in this research with other polymer research centers in Sofia, Bulgaria (Institute of Polymers of the Bulgarian Academy of Sciences), Dresden, Germany (Leibniz Institute of Polymer Research and Technical University), Bratislava, Slovakia (Polymer Institute of the Slovak Academy of Sciences), Leuven, Belgium (University of Leuven) and Łódź, Poland (CMMS PAS).

In recent years, research has also been oriented toward thermoresponsive polymers in the form of polymer layers and thin films. Establishing cooperation with the Institute of Applied Radiation Chemistry of the University of Technology in Łódź and the Center for Burn Treatment in Siemianowice Śląskie and the Medical University of Silesia in Katowice led to the development of thermoresponsive surfaces for skin cell culture and gene therapy and to surfaces with antifouling and antimicrobial properties.

**Figure 1:**
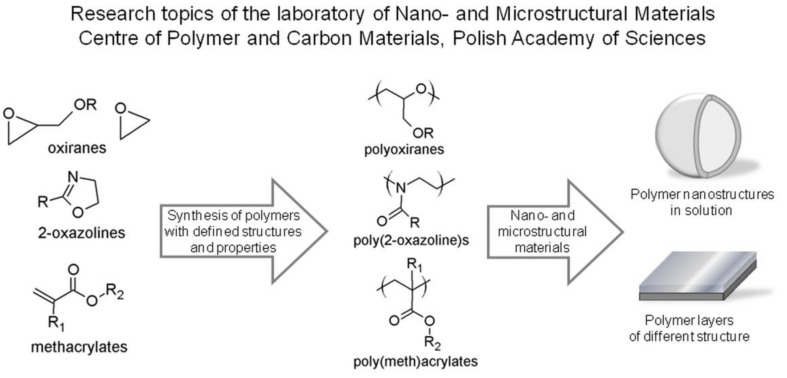
Schematic representation of the monomers, polymers and nano- and microstructural materials obtained in the Laboratory of Nano- and Microstructural Materials of CMPW PAS.

The investigations of the living and controlled polymerization processes of cyclic oxiranes, 2-oxazolines and methacrylates carried out by the group of Prof. Dworak were pioneering and led to obtaining more complex structures, for example, bottle brushes, dendritic structures, stars, etc. The obtained biocompatible and smart (co)polymers were of interest to the scientific community for many years with the aim of their application in medicine and biology, in agreement with global trends [6,7,8,9]. Currently, the research topics of the Laboratory of Nano- and Microstructural Materials focus on the study of the phenomena of the organization of macromolecules in solution, especially thermoresponsive and amphiphilic polymers, hybrid formation of polymer and lipids and nucleic acids and the synthesis of new polymers with complex macromolecule architecture for biomedical applications. The present research topics of the laboratory broaden the known studies on polymers based on oxiranes, cyclic imines and methacrylates (Figure 1).

This review summarizes the investigations carried out in the Laboratory of Nano- and Microstructural Materials at the Centre of Polymer and Carbon Materials, PAS for over 30 years. We hope that the achievements presented here will allow the reader to locate and compare the work and the topics with the general scientific literature [10,11,12,13]. We believe that studies started by Prof. Andrzej Dworak and continued by his team are essential for understanding the principles of synthesis of polymers with desired properties by the control of the polymerization process. These studies are crucial for designing polymers for different applications. Our studies make an important contribution to strengthen the research carried out in the field of polymer chemistry and nanotechnology, and are of importance in the progress of the development of nano- and microstructural materials with the desired perspective, mainly in bioapplications.

## 2. Polymer Nano- and Microstructure

### 2.1. Polymer Nanostructure in Solution

Polymer nanostructures can be defined as systems with diameters ranging between 1 and 1000 nm. They can exhibit great morphological diversity, forming spheres, vesicles, cylinders, disks, hollow spheres, tubes or nanolayers. Their properties, such as stability, size, shape, surface charge, surface chemistry, mechanical strength, porosity and functionality, can be tailored to match targeted applications.

**Figure 2:**
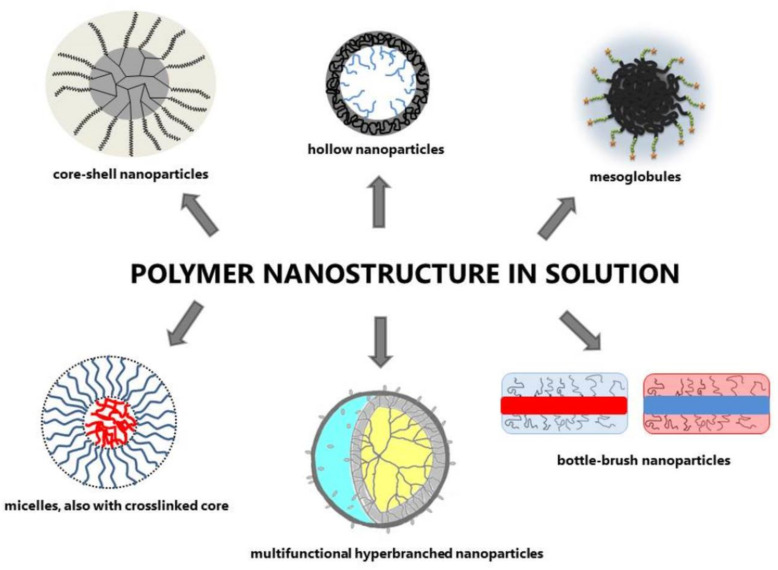
Schematic representation of the polymeric nanostructures in solution obtained in the Laboratory of Nano- and Microstructural Materials of CMPW PAS.

In the Laboratory of Nano- and Microstructural Materials, different kinds of polymer nanostructures in solution have been obtained, and they are schematically presented in Figure 2.

#### 2.1.1. Mesoglobules of Thermoresponsive Polymers

Thermoresponsive polymers, because of their possibility to self-aggregate, were used in our laboratory to create polymer nanostructures. In dilute aqueous solutions, above a certain temperature called the cloud point temperature (T_CP_) or phase separation temperature, the polymers frequently form mesoglobules—colloidally stable, equally sized (with diameters ranging from tens to hundreds of nanometers) spherical aggregates formed by collapsed, dehydrated polymer chains. The sizes of mesoglobules are determined by the polymer concentration [14,15,16], molar mass [15], composition of the (co)polymer [17,18], heating procedures of solutions [16,19] or the presence of additives. The process of mesoglobule formation is reversible, and after lowering the temperature, the particles disaggregate. Such behavior limits their potential applications. Therefore, we have stabilized the mesoglobules by covering them with a crosslinked shell [17,20,21,22,23,24] or by crosslinking the collapsed polymer chains [25,26,27].

In our laboratory, several kinds of thermoresponsive polymers, such as copolymers of oligo(ethylene glycol) methacrylates (POEGMAs) [16,28,29,30], poly[oligo(ethylene glycol) acrylates] (PETEGAs) [21,31], copolymers of oligo(ethylene glycol) methacrylates with 2-aminoethyl methacrylate hydrochloride (P(D-co-A_A)), with prop-2-yn-1-yl carbamate methacrylate (P(D-co-A_Pr) and 2-azidoethylmethacrylate (P(D-co-A_Az)) [25,26,27], poly(N-isopropylacrylamide) (PNIPAM) [20], poly(2-isopropyl-2-oxazoline) (PIPOx) [22] and hydrophobically modified polyglycidol (poly(glycidol-co-ethyl glycidyl carbamate), mPGL) [17], were used in the process of mesoglobule formation. The results are summarized in Table 1. We have also studied the aggregation behavior of thermoresponsive polymers conjugated with pentapeptide glycine–arginine–lysine–phenylalanine–glycine–dansyl (GRKFG-Dns) [32,33] or met-enkephalin [24,28].

A detailed analysis of the influence of the molar mass, copolymer composition, initial polymer concentration and protocol for mesoglobule formation (gradual, abrupt heating and dropwise addition, also called nanoprecipitation) on the mesoglobule properties was performed.

The research data indicated that, regardless of polymer type, the slow, gradual heating method produced large mesoglobules even above 1 µm (Table 1). This results from the association of polymer chains with each other before they collapse. Abrupt heating leads to the formation of well-defined mesoglobules with considerably smaller dimensions and a narrow size distribution, because fast heating leads to more pronounced intrachain contraction and less interchain association. The heating of polymers at temperatures far above their cloud point temperature resulted in a decrease in the mesoglobule dimensions, and their size distribution became more defined [31]. A so-called nanoprecipitation protocol was used to obtain PIPOx and mPGL mesoglobules [17,22]. It consisted of dropwise addition of aqueous polymer solution of a certain concentration and temperature below the T_CP_ to a certain volume of water preheated to temperatures above the polymer T_CP_. Such a procedure led to well-defined particles and further reduced their sizes, but what is important is that the size reproducibility was considerably improved.

Regardless of the type of thermoresponsive polymer, the mesoglobule sizes decreased with decreasing (co)polymer concentration and with decreasing molar mass (Table 1).

In the case of copolymers, by changing the hydrophilic to hydrophobic balance in a chain, the effectiveness of aggregation can also be altered. For our POEGMA copolymers, the size of the mesoglobules decreased with increasing length of hydrophilic OEG chains and with their increasing content in the copolymer [16]. It was suggested that the hydrophilic OEG side chains were placed at the periphery of the mesoglobules and were in direct contact with the surrounding water, thus controlling the size of mesoglobules as well as their stability. POEGMA aggregated into loose mesoglobules contains a significant amount of water. For a copolymer series P(D-co-A_A), P(D-co-A_Pr) and P(D-co-A_Az) (the same amount of amine, propargyl and azide groups in copolymer, respectively), the mesoglobule sizes decreased with increasing comonomer affinity to water [25,26]. Copolymers with amine and azide groups, relatively hydrophilic groups, formed well-defined mesoglobules whose sizes decreased with the increase in their amount in the copolymer chain. This was probably due to the concentration of these hydrophilic groups on the surface of the collapsed particle, which reduced the contact of the aggregates and thus their sizes [25]. The sizes of particles made from copolymers with hydrophobic propargyl groups are significantly larger than those obtained under the same conditions for their amine or azide counterparts. Slightly different behavior was observed for mPGL with different contents of hydrophobic ethyl carbamate groups [17]. The aggregates formed by more hydrophilic polymers, containing larger amounts of hydrophilic -OH groups, were of a loose structure, swollen by the water molecules and thus of larger sizes than those formed by more hydrophobic polymers [17].

**Figure 3:**
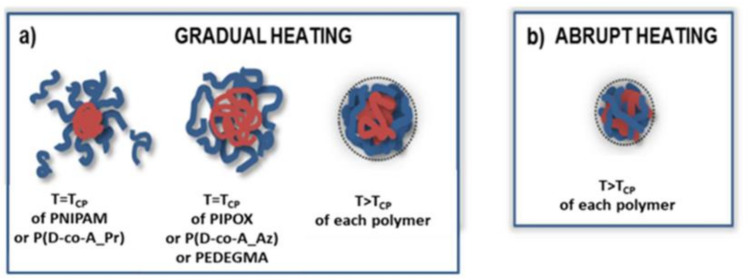
Schematic presentation of the strategy for preparing mesoglobules in mixtures of thermoresponsive polymers: (**a**) with a core–shell structure and (**b**) homogenously mixed chains.

In our lab, mesoglobules obtained from mixed thermoresponsive copolymers were also studied [23,25,26,28]. During gradual heating of the PNIPAM/PIPOx mixture, 3 transitions were distinguished: the two low-temperature transitions were correlated with the T_CP_ of the individual polymers, whereas the high-temperature transition was associated with the polymer mixture and was composition dependent [23]. With larger amounts of PIPOx in the mixture, PIPOx crystallization was observed, which led to a constant increase in the mesoglobule sizes with time. The morphology of the obtained particles was composition dependent, and crystalline nanofibers interconnected to the mesoglobules were visible for PIPOx-rich systems. Separate transitions appeared in transmittance curves for individual components in P(D-co-A_Pr)/P(D-co-A_Az) or PNIPAM/POEGMA mixtures [25,28]. The size of mesoglobules was controlled by changing the total concentration and the ratio of the polymers in the mixed solutions. In gradually heated mixtures of thermoresponsive polymers, the obtained particles had core–shell structures. The core was formed by a low transition temperature polymer, and the shell was formed by a polymer of higher T_CP_ (Figure 3a). Although the particle size distributions of the formed mesoglobules were relatively narrow, they were characterized by large dimensions (diameters reaching from 300 nm to 1 µm) [23,25]. In contrast, abrupt heating resulted in the formation of better defined mesoglobules with considerably smaller diameters (<200 nm) [23,25]. When the solution was abruptly heated to a temperature that was well above the T_CP_ of the individual copolymers, mesoglobules were formed in one step. Thus, they were composed of strongly entangled and intertwined chains of the two polymers (Figure 3b).

It was observed that the sizes of mesoglobules can be altered by salt or surface-active agents present in polymer solution (Table 1). The presence of the anionic surfactant SDS caused a 50% decrease in PNIPAM mesoglobule sizes [20,28], 80% for POEGMA nanoparticles [28] and 20% for a PNIPAM/POEGMA mixture [28]. The influence of the additives was also dependent on the molar mass of the polymer. The mesoglobules formed by PIPOx of a higher molar mass have their sizes significantly reduced by the presence of SDS [22]. Upon the addition of SDS to a solution of PETEGA [21,31], smaller aggregates were formed at the respective temperature for polymers of higher molar mass, and the increase in temperature led to a further decrease in the sizes. However, when the SDS content increased, the aggregates became smaller, but a bimodal distribution was observed. Nevertheless, by applying an appropriate solution composition and S/P ratio (surfactant to polymer ratio), it was possible to control the size and size distributions of nanoparticles. A different dependence was observed for the copolymers of glycidol and ethyl glycidyl carbamates with different contents of hydrophobic ethyl carbamate groups [17]. The addition of SDS or cationic CTAB surfactant to the solution of copolymer with fewer hydrophobic ethyl carbamate groups resulted in a significant increase in the mesoglobule sizes. The reason is likely that the loose interior of aggregates formed by such a copolymer is easily accessible to the surfactant molecules, leading to the expansion of the mesoglobules by the repulsive interaction of the surfactant head groups. The phenomenon is considerably stronger for CTAB. In the case of the copolymer with more hydrophobic ethyl carbamate groups, the mesoglobules formed with SDS are larger than those formed from the pure copolymer solution only at low copolymer concentrations. As the copolymer concentration increases, the mesoglobule size decreases drastically.

In our laboratory, mesoglobules were also prepared from conjugates of thermoresponsive polymers with peptides and drugs. The conjugation of peptides or drugs with synthetic polymers is an important trend in polymer chemistry, as it opens a route to nanocarriers and to control drug delivery to target sites. For that purpose, pentapeptide (met-enkephalin or GRKFG-Dns) macroinitiators were prepared and then used for (co)polymerization of oligo(ethylene glycol) methacrylates or N-isopropylacrylamide by the ATRP technique [24,28,32,33]. The sizes of the mesoglobules were controlled by selecting the heating rate and concentration of the bioconjugate solution and by the presence of SDS. The aggregates made of PNIPAM or POEGMA conjugated with GRKFG-Dns consisted of a core formed by partially dehydrated, collapsed thermoresponsive polymer chains surrounded by a thin corona formed by the attached peptide chains. Such a structure allowed the peptide segments in mesoglobules to be easily released by enzymes [32,33]. To prepare the drug nanocarriers, doxorubicin (DOX) was covalently bound to an oligo(ethylene glycol) methacrylate (co)polymer chain via carbamate linkage [27]. Next, thermal coaggregation of the polymer (containing azido groups)–DOX conjugate and its polymer partner (containing propargyl groups) was performed above the T_CP_ of both partners, leading to mixed mesoglobules. A click reaction was used to crosslink the chains. In this way, stable, spherical nanocarriers were obtained [25,27]. The disintegration of nanocarriers and the detachment of DOX took place as a result of hydrolysis of the carbamate bonds. The addition of the enzyme accelerated this process.

**Figure 4:**
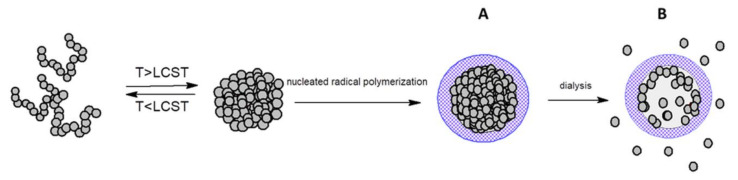
Nanoparticles based on mesoglobules with crosslinked shell (**A**) and hollow interior (**B**).

Another way to stabilize mesoglobules is covering them with crosslinked shell [17,20,21,22,23,24]. For that purpose, mesoglobules obtained from mPGL, PETEGA or mixed PIPOx/PNIPAM were used for nucleated polymerization of NIPAM or hydroxyethyl methacrylate (HEMA) with *N*,*N*′-methylenebisacrylamide as a crosslinker [17,21,23] (Figure 4, structure A). The nanoparticles were larger than the mesoglobules used, indicating that core–shell structures were obtained. The shell thickness was approximately 20–30 nm. Upon cooling to temperatures below the T_CP_ of the components used for the formation of stable mesoglobules, the resulting nanoparticles swelled significantly. Interestingly, the nanoparticles repeatedly, reproducibly and quickly responded to the temperature by changing their dimensions [17,23].

For nanoparticles composed of the PNIPAM or PIPOx core and PHEMA or PNIPAM shell, respectively, hollow spheres were obtained (Figure 4, structure B) [20,22] by subjecting the nanoparticles with a crosslinked shell to extensive dialysis against water cooled to a temperature much lower than the T_CP_ of the core components. It was possible to remove more than 42% of PNIPAM [20] and more than 80% of PIPOx from the interior of the nanoparticles [22]. The process of core removal was facilitated by the use of a low molar mass polymer and by reducing the crosslinking density of the shell.

An interesting and novel approach to polymeric nanocarriers using mesoglobules was recently presented in [24]. A mesoglobule of POEGMA bioconjugate with met-enkephalin was covered by a PNIPAM two-layer shell, followed by functionalization with a targeting moiety—RGD with a fluorescent label. The presence of degradable disulfide bonds in the crosslinked shell allowed for disintegration of the nanoparticles with glutathione and release of the bioconjugate. Such a strategy of the formation of precisely engineered nanoparticles protecting their cargo, based on mesoglobules, constitutes a new pathway for targeted therapy.

#### 2.1.2. Branched Nanostructures

##### Hyperbranched Polymers

The synthesis of branched or hyperbranched polymers requires monomers that form more than two linkages during the polymerization process. Copolymerization with the other two functional monomers allows a less dense than hyperbranched structure to be obtained. Knowledge gained from previous research conducted in the laboratory concerning the mechanisms of polymerization of branching monomers, glycidol and glycidol with protected hydroxyl groups (dormant branching site) [34,35,36,37] and p-(iodomethyl)styrene [38], was applied for the synthesis of branched-chain macromolecules.

In our laboratory, glycidol was of particular importance in the synthesis of branched polymers. After appropriate blocking of the hydroxyl group, preferably with an acetal group, it polymerizes according to an anionic mechanism in a controlled manner [36]. After polymerization, protected hydroxyl groups can be hydrolyzed, and linear polyglycidol is obtained (PGL). The hydroxyl groups in the polymer can serve as branching points and thus allow the synthesis of branched polymers and copolymers with glycidol branching.

Arborescent-branched grafted glycidol polymers were synthesized by multiple processes of anionic polymerization of glycidol with a protected hydroxyl group (1-ethoxyethyl glycidyl ether, EEGE), hydrolysis of the acetal groups in the resulting linear polymer and then using the obtained polyglycidol as a macroinitiator for subsequent EEGE grafting [39]. The ionization of the hydroxyl groups of the macroinitiator was approximately 8% of the total hydroxyl groups in each step of the synthesis, while the fraction of linear units grafted in every single step of grafting was between 70 and 90%. This means that the proton exchange between the alcohol and alkoxy groups in this process is rapid and enables chain growth at approximately 10 times the amount of the initially ionized hydroxyl groups. The grafting process was repeated three times. In the first and second steps, the degree of branching was below 20%, while after the third grafting, it was already above 60%. The dendritic polyglycidol finally obtained has a molar mass exceeding 1.8 × 10^6^ g/mol and a dispersity of 1.43.

Grafting of linear polyglycidol chain units was also used to synthesize high molar mass, arborescent copolymers of ethylene oxide with a pom-pom structure [40] and copolymers of a dendritic star structure [41]. In the first case, linear poly(ethylene glycol) (PEG) with two ionized hydroxyl end groups served as the macroinitiator for the polymerization of EEGE of low DP (from 5 to 6). The hydrolysis of acetal groups resulted in a block copolymer, which was used for the polymerization of ethylene oxide. After completion, the EEGE monomer was added to form the next short glycidol blocks. In the multistep synthesis consisting of two polymerizations of ethylene oxide, the final copolymer has a structure of two stars linked by the oxyethylene chain and arms formed by dendritic oxyethylene chains with many hydroxyl end groups originating from short polyglycidol blocks. The molar mass of the final copolymer was over 200,000 g/mol, and the dispersity was very low at 1.03. The obtained arborescent polymers were highly biocompatible toward different lines of cells and DNA [42].

A similar method of synthesis was applied to obtain poly(ethylene oxide) (PEO) stars using tetrafunctional pentaerythritol instead of a linear polymer in the first step [41]. Dendritic stars with different block lengths between branches, molar masses up to 60,000 g/mol and dispersion not exceeding 1.05 were obtained. It was found that T_m_ values increase with PEO block length and are significantly lower than those for homopolymers of ethylene oxide with similar DPs.

Optimal conditions for the well-controlled radical polymerization of p-(iodomethyl)styrene, a monomer which contains two groups potentially active in radical polymerization, have been developed [38]. The application of AIBN as an initiator instead of peroxides and a moderate temperature near 67 °C led to fully soluble products. It was found that the molar mass of the obtained branched poly[p-(iodomethyl)styrene] increased with monomer conversion and can be easily controlled by the polymerization time.

##### Bottle-Brush (Comb-Like) Macromolecules

The living character of the polymerization of glycidol acetal initiated with metal alcoholates made it possible to synthesize a reactive macromonomer [36,43,44,45]. For that purpose, the polymers of glycidol acetal or block copolymers of glycidol acetal with glycidyl phenyl ether were terminated with p-(chloromethyl)styrene. After hydrolysis of the protecting glycidol groups, well-defined hydrophilic [36,43] and amphiphilic [44,45] macromonomers with a lipophilic, polymerizable styrene unit were obtained. The degree of polymer chain functionalization was estimated to be 0.65–0.8.

The hydrophilic polyglycidol macromonomers during emulsifier-free copolymerization with styrene yielded microspheres enriched with hydroxyl groups on the surface [36,43]. The diameters of the particles (ranging from 200–900 nm) decreased with an increasing fraction of polyglycidol macromonomer and with its molar mass. It was observed that the hydrophilic, hairlike polyglycidol chains on the surface of the microspheres efficiently protected the microstructure from protein adsorption.

Amphiphilic poly(glycidol-b-glycidyl phenyl ether) macromonomers with a styrenic group attached to either a hydrophobic or hydrophilic block were radically homopolymerized in water or in a water/benzene mixture, leading to cylindrical bottle-brush macromolecules with high molar masses [44,45]. The homopolymerization of macromonomers was fast, and the conversion of macromonomers reached 90–99%. In the polymerization mixture, the macromonomers created micelles and aggregates with different structures. The obtained polymacromonomers, in contrast to macromonomers used for their synthesis, did not show amphiphilic properties. The solubility of polymacromonomers depends on the properties of the surrounding outer shell of the polymacromonomer (Figure 2).

##### Stars

**Figure 5:**
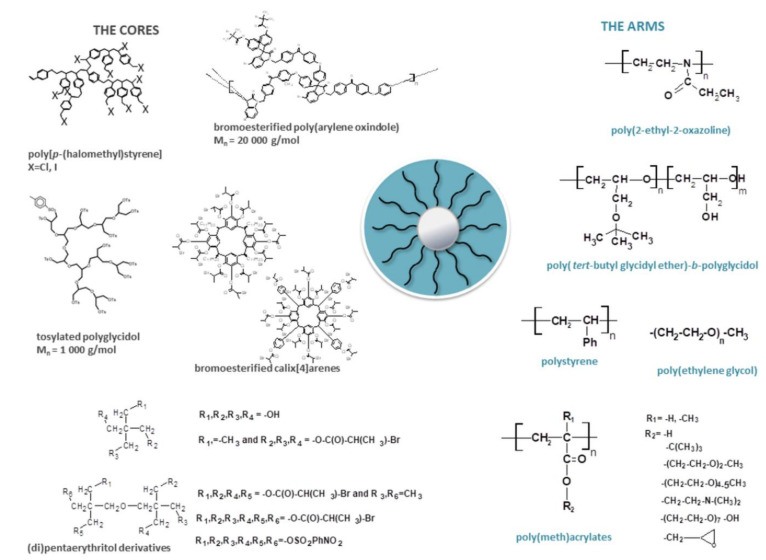
Structure of the studied star polymers.

Another group of polymers studied in our laboratory are polymers of star topology. Different kinds of stars were obtained during the last two decades, both with cores made of low molar mass compounds [46,47,48,49,50,51] and with highly branched polymers [38,46,52,53,54,55,56,57,58,59,60,61,62,63]. The arms of the stars were formed by linear homopolymers and/or copolymers of various architectures (block, random, etc.). A schematic presentation of the star cores and their polymer arms is shown in Figure 5, while all the synthesized stars and information on their detailed application are summarized in Table 2.

Living polymerization methods (cationic and anionic), atom transfer radical polymerization (ATRP) and iodine-mediated degenerative chain transfer polymerization (IDT) have made it possible to control the topology of the polymers (number and length of the arms of stars) and to obtain low dispersity of their molar masses.

The use of the “arm-first” method (termination of living linear chains by a multifunctional terminating agent) enabled us to obtain star polymers with a hyperbranched poly[p-(chloromethyl)styrene] core and poly(ethylene glycol) arms. The structures were synthesized as a result of the Williamson etherification reaction between active halogen groups of branched polystyrene and living linear polyether macroanions [52].

Then, we focused mainly on the synthesis of stars by the “core-first” method, where the multifunctional core groups initiated the polymerization of the monomer, leading to the formation of star arms. The advantage of this method was the absence of linear unreacted chains in the obtained product. The synthesis of stars with various cores and polystyrene [38,53], poly(meth)acrylates [50,51,54,56,57,58,59,60,61,62], polyacid [55,63], polyether [47,48,49,52] and polyoxazoline [46] arms led to stars with designed parameters, including the number and length of arms, the molar mass and low dispersity.

Indirect proof that the polymerization was well controlled was the estimation of the real functionality of the star polymers. This was carried out for poly(tert-butyl methacrylate) (PtBuMA) stars [62], poly[di(ethylene glycol) methyl ether methacrylate-co-oligo(ethylene glycol) methyl ether methacrylate] (P(DEGMA-co-OEGMA)) stars [56] and poly(N,N’-dimethylaminoethyl methacrylate) (PDMAEMA) stars [57] by selective alkaline hydrolysis of the ester linkages between the core and arms. The number of arms was calculated based on the molar mass of the star before and after hydrolysis. For PtBuMA and PDMAEMA stars, the number of arms is close or even the same as the number of initiating sites in the hyperbranched core [57,62]. For P(DEGMA-co-OEGMA) stars, the calculated functionality was lower [56], which indicates limited access of monomer to the initiating site.

The behavior of branched macromolecules in solution is very complex and depends on the kind of arms, their lengths and composition and the solvent used. The relationship between these parameters and the solution features of synthesized macromolecules was of great importance.

The star structure of the polymers proven by its hydrodynamic properties has been thoroughly investigated by GPC-MALLS. The dimensions (the hydrodynamic volumes) occupied by star macromolecules in solution were found to be smaller in comparison with the linear chains of the same molar mass. For polyoxazoline [46], poly(t-butyl acrylate) (PtBuAc) [50,51,61] and P(DEGMA-co-OEGMA) [56], star branching parameters were calculated. Four-arm PtBuAc stars with molar masses greater than 100,000 g/mol were tested by static and dynamic light scattering methods. This allowed us to obtain scaling equations for the radius of gyration, hydrodynamic radius, second viral coefficient and diffusion coefficient for the studied PtBuAc stars in a good solvent [50,51].

The majority of synthesized star (co)polymers are composed of hydrophobic cores and hydrophilic arms. This fact influenced the solution behavior and more specifically favored the aggregation of stars.

Generally, in organic solvents, good for both arms and the core, star polymers occur as isolated macromolecules. Such behavior was observed in acetone for stars with a hydrophobic poly(arylene oxindole) (PArOx) core and P(DEGMA-co-OEGMA) [56], PDMAEMA [57,59], P(DMAEMA-co-DEGMA) [58] and P(DMAEMA-co-OEGMA-OH) arms [60]. The sizes of star nanostructures in aqueous solutions were found to be in the range of several tens of nanometers, which was desirable for their future use in medicine.

Changing the solution temperature affected the organization of several types of star macromolecules described above. Polyoxazoline, poly(tert-butyl glycidyl ether)-b-polyglycidol, P(DEGMA-co-OEGMA) and stars with polycationic arms were thermoresponsive [46,47,48,56,57,58,60,64]. PDMAEMA stars were thermoresponsive in water only at pH ≥ 13 [57], while for P(DMAEMA-co-OEGMA) stars (not thermoresponsive in water), an abrupt response to temperature appeared in the culture medium at 34 °C [65]. For polyoxazoline [46] and PDMAEMA [57] stars, increasing length of the arms reduced the transition temperature. The transition temperature of star copolyethers depended on the ratio of hydrophobic poly(tert-butyl glycidyl ether) to hydrophilic polyglycidol blocks and increased with the length of the latter. The transition temperature also increased with increasing hydrophilic block number in the outer layer [48]. In the case of stars with P(DMAEMA-co-OEGMA-OH) arms, T_CP_ values were dependent on the ratio of comonomers in the arm [60]. T_CP_ increased with hydrophilic OEGMA-OH units in the arms up to 10 mol%, while for the stars with an OEGMA-OH content of 10 mol%, their thermoresponsiveness was switched off [60]. Polyether stars formed aggregates with hydrodynamic diameters on the order of several nanometers below the phase transition temperature [48]. However, P(DMAEMA-co-OEGMA-OH) stars remained isolated below the transition and aggregated above it [60]. Poly(acrylic acid) (PAA), poly(methacrylic acid) (PMA) and PDMAEMA stars show pH responsiveness [55,57,59,63]. Research has indicated that the most important property for the behavior of star polymers with PAA arms in aqueous solution is the size of the hydrophobic interior and the number of star arms forming a pH-sensitive shell. Stars with a large hydrophobic poly(arylene oxindole) core and polyelectrolyte arms aggregate in water in a pH-dependent manner, while polyacid stars with a smaller poly[p-(iodomethyl)styrene] core and PAA arms are present as single stars or small aggregates of multiple stars. The behavior of these structures in solutions is strongly dependent on the composition, molar mass and number of arms of the stars.

In recent years, research into star-shaped polymers has evolved toward specific bioapplications. Therefore, it is important to determine their cytotoxicity in the concentration ranges used in biological tests. PDMAEMA, P(DMAEMA-co-DEGMA) and P(DMAEMA-co-OEGMA-OH) stars have been shown to be nontoxic to HT-1080 human fibrosarcoma cells [57,58,60], while P(DEGMA-co-OEGMA) stars are nontoxic to fibroblasts [65]. Stars with a poly[p-(iodomethyl) styrene)] core and poly(acrylic acid) arms were not neurotoxic and did not induce an immune response in the organism [66].

Stars with a poly[p-(iodomethyl) styrene)] core and poly(acrylic acid) arms have been used for conjugation with cisplatin [66], which is a drug used in cancer chemotherapy. A very high loading efficiency of the drug, up to 80%, corresponds to approximately 45% by weight of platinum in the loaded macromolecules. The release profiles of the drug complexes have shown that the drug is released continuously from the complex; approximately one-third of cisplatin is released within nine days [55]. Polymers containing an additional outer layer of poly(ethylene glycol) have higher loading efficiency and release more drug for a longer time than stars that do not contain PEG chains [67]. Solutions and suspensions of red blood cells containing stars with arms of poly(acrylic acid) have a higher apparent viscosity and a lower electrical conductivity than solutions that contain linear analogs [68].

Stars containing cationic PDMAEMA arms were used to complex nucleic acids into so-called “polyplexes”. Such complexes are promising carriers of DNA and RNA in gene therapy [69]. The sizes, zeta potential and shape of the obtained complexes were determined in addition to the biological experiments (cytotoxicity and transfection) performed on the HT-1080 cell line for PDMAEMA, P(DMAEMA-co-DEGMA) and P(DMAEMA-co-OEGMA-OH) star polymers [57,58,59,60,70]. Poly(ethylene glycol) methacrylate was introduced into the arms to decrease the cytotoxicity of the PDMAEMA stars and their polyplexes. The created complexes were capable of facilitating the uptake of therapeutic nucleic acids and preventing their degradation in human fibrosarcoma HT-1080 cells [57,58,59,60,70]. The star (co)polymers exhibited significant nucleic acid delivery, depending upon the length of the arms and their chemical composition (oligo(ethylene glycol) methacrylate content) and the structure of the arms (block, random). The cytotoxicity of polyplexes was kept low due to the presence of OEGMA units in their structure [58,59,60,70].

The cationic PDMAEMA star and its linear analog were able to reduce silver ions and led to the formation of silver nanoparticles (AgNPs) without any additional reducing agent. The polymer acts simultaneously as a stabilizer of the formed nanoparticles [71]. The hybrid nanomaterial based on star PDMAEMA was more stable than AgNPs with linear PDMAEMA, which indicated that the star structures better prevented undesirable aggregation of silver nanoparticles in the solution. The hybrid nanomaterials were investigated as antimicrobial agents against three strains of bacteria: Bacillus subtilis, Escherichia coli and Pseudomonas aeruginosa. The research was then extended to quaternized and nonquaternized star and linear polymers. All studied structures exhibited antibacterial activity against the tested bacterial strains, but the incorporation of AgNPs into polymer structures enhanced the bactericidal activity of such hybrids by several times [71].

The stars with the P(DEGMA-co-OEGMA) arms were investigated as nanocarriers of the model fluorescent compound 4-(dicyanomethylene)-2-methyl-6-(4-dimethyl aminostyryl)-4H-pyran [56]. It was shown that the star loaded with a hydrophobic dye as an encapsulated guest does not aggregate at room temperature up to concentrations of 1 g/L, which makes these “core–shell” structures promising candidates for application in controlled drug delivery systems.

#### 2.1.3. Nanostructure via Self-Assembling of Block Copolymers

The presence of hydrophilic and hydrophobic segments in amphiphilic block copolymer chains causes their self-assembly when dissolved in a selective solvent that is thermodynamically good for one block of the copolymer and poor for the second block. Aggregation can also be induced in the case of so-called double hydrophilic copolymers with one stimulus-responsive block. When an external stimulus is applied, such a block undergoes a transition from soluble to insoluble in water, which induces the aggregation of the copolymer into nanostructures. The nanostructures obtained from both amphiphilic and double hydrophilic copolymers are stable only above critical conditions, e.g., concentration or temperature. Therefore, many potential commercial applications of micelle-based polymer nanomaterials are significantly limited due to their instability under changeable environmental conditions. To improve micelle stability, crosslinking of their core or their shell is often applied, leading to nanogels.

The methods of ionic polymerization of oxiranes and oxazolines, developed in our laboratory, allowed us to obtain a series of well-defined di- and triblock amphiphilic copolymers with the general structures of AB, ABA and BAB, where A is a hydrophilic block, poly(ethylene oxide), polyglycidol or poly(2-ethyl-2-oxazoline), and B is a hydrophobic block made of hydrophobically substituted polyglycidol (with ethyl, n-propyl, n-butyl, n-pentyl carbamate groups or cinnamic groups), polystyrene or poly(2-phenyl-2-oxazoline) [73,74,75,76,77,78]. The AB copolymers, where A is a hydrophilic polyglycidol block and B is a thermoresponsive PNIPAM [79,80,81] or pH-responsive poly(4-vinyl pyridine) (P4VP) block [82], were obtained using the combination of controlled anionic and radical polymerizations. The prepared copolymers assembled into nanostructures under certain conditions.

**Figure 6:**
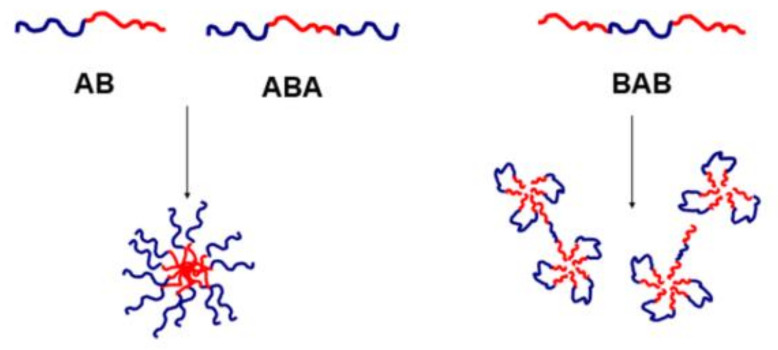
Schematic representation of AB, ABA and BAB copolymers of ethylene oxide (A block) and hydrophobically modified glycidol (B block) aggregates in water.

Block copolymers of PEO (A) and polyglycidol modified with alkyl groups of different lengths (B) form in water-aggregated structures, the arrangement of which depends on the architecture of the macromolecules. ABA- and AB-type copolymers self-assembled into micelles with hydrophobic blocks as a core, and hydrophilic blocks created the shell (Figure 6). For copolymers with ethyl groups, no cloud point was observed in the case of AB architecture, and the T_CP_ for ABA copolymers was approximately 80 °C [73]. The T_CP_ of ABA copolymers decreased to 65 °C with increasing length of the hydrophobic alkyl group [74]. In the case of the BAB triblock copolymer, several structures could be obtained: the so-called “flower like” structure, in which central block A adopts a loop conformation and block B forms the micelle core, and aggregates formed by combined micelles and intermediate structures (Figure 6). The T_CP_ of this polymer (46 °C) was thus much lower than the clouding temperature of the copolymer of similar chemical composition but with ABA architecture [73].

Temperature also played an important role in the process of reorganization of the aggregates [73,74]. For AB and ABA copolymers, for a highly hydrophobic unit content and length of the n-alkyl hydrophobic chain, monomodal nanostructures were formed at a lower temperature, and a temperature increase did not significantly influence their R_h_ values. For these structures, the radii of gyration, molar masses, aggregation numbers and shape (spherical and rod-like structures) were also dependent on the hydrophobic unit content and the length of the n-alkyl chain [74]. For BAB copolymers, the size distribution was bimodal [73]. The smaller aggregates were attributed to micelles, while the loose, larger aggregates were presumably of a nonmicellar nature. At an elevated temperature, the micelles were reorganized, and a well-defined nanostructure of a hydrodynamic radius (R_h_) of 5–15 nm was obtained.

With the cooperation of the CMMS PAS in Łódź, the self-assembly of amphiphilic polystyrene-b-polyglycidol copolymers (PS-b-PGL; the same hydrophobic block length and various numbers of glycidol units in the hydrophilic block) was investigated [77,78]. The formation of nanostructures in water after dialysis from organic solvents (dioxane and dimethylformamide) [77] and in a dioxane/water mixture [78] was followed. The morphology of the aggregates strongly depended on the initial solvent. When the polymer was dialyzed from dimethylformamide, particles of sizes from 20 to 250 nm were created, which indicated that not only typical small core–shell objects but also large copolymer/micelle clusters were formed. Uniform and spherical core–shell micelles appeared when the polymers were dialyzed from dioxane. Dioxane is a good solvent only for polystyrene and, thus, a polyglycidol block collapses and is surrounded by polystyrene. This promoted the formation of well-defined regular micelles with PS cores during dialysis to water. These micelles were extremely stable even when diluted 10 times below the critical micelle concentration (CMC). Particle formation for PS-b-PGL copolymers was also performed by the addition of water to the copolymer solution in dioxane. The copolymer self-assembled above the critical water content into monomodal nanoparticles with diameters of 30–325 nm depending on the PGL/PS ratio and the initial copolymer concentration.

Copolymer composition was observed to have a great influence on the aggregation process and formation of nanocolloidal systems for amphiphilic diblock copolymers of 2-ethyl-2-oxazoline and 2-phenyl-2-oxazoline [76]. The nanostructures with R_h_ between 60 nm and 235 nm obtained via the solvent exchange procedure were larger than the core–shell type micelles. Upon increasing the length of the hydrophobic chain, the concentration range of the formation of stable nanocolloidal solutions shifted to lower values, and the stability of nanoparticles increased. Copolymers with almost equal mass fractions of hydrophobic and hydrophilic blocks formed stable micelles (of approximately 20 nm) that self-assembled into aggregates that dissociated into core–shell micelles upon application of mechanical shear.

In cooperation with Technical University in Dresden, AB block copolymers of polyglycidol with thermoresponsive PNIPAM or pH-responsive P4VP segments were obtained via a macroinitiator technique [79,80,81,82]. The aggregation of these polymers under the influence of temperature or pH led to aggregates of R_h_ 70–180 nm, depending on the block composition and content. It was possible to obtain stable, core–shell micelles with compact cores formed from collapsed stimulus-responsive segments stabilized by the surrounding hydrophilic polyglycidol shell.

We have shown the possibility of stabilizing well-defined, small monodisperse nanoparticles via UV irradiation [75,81]. The diblock copolymer of poly(ethylene oxide) and polyglycidol modified with cinnamic acid, which generated the amphiphilic character of the block, was synthesized [75]. The chains spontaneously self-associated in aqueous solution into micelles of R_h_ 9.2–12 nm with poly(ethylene oxide) corona and a poly(glycidol-co-glycidyl cinnamate) core. Additionally, well-defined diblock copolymers of PGL/PNIPAM with randomly localized dimethyl maleimido chromophores in a PNIPAM segment were prepared [81]. In both cases, the micelle cores in a pure, initiator-free water system were crosslinked by using UV irradiation. All nanomaterials were stable and did not precipitate after a long storage time, which is of great importance in regard to their potential applications.

### 2.2. Polymer Layers of Different Structures

**Figure 7:**
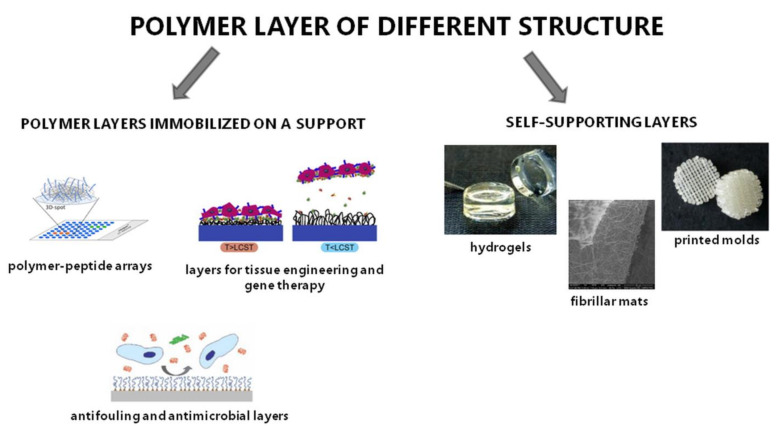
Schematic representation of the polymer layers obtained in the Laboratory of Micro- and Nanomaterials of CMPW PAS.

Our aim was also to develop and compare new biocompatible polymer layers with different compositions and structures and to determine the relationship between the properties of the surfaces covered with polymers and their interaction with biologically active substances. Such analysis was essential to define the potential use of the obtained materials in reconstructive medicine and tissue engineering. Polymer coatings were obtained in the form of self-supporting layers such as hydrogels or matrices in the form of nonwoven fibrous mats and three-dimensional molds and as layers immobilized or adsorbed on a support. The general scheme presenting the idea of the performed work is presented in Figure 7.

#### 2.2.1. Polymer Layers Immobilized on a Support

**Figure 8:**
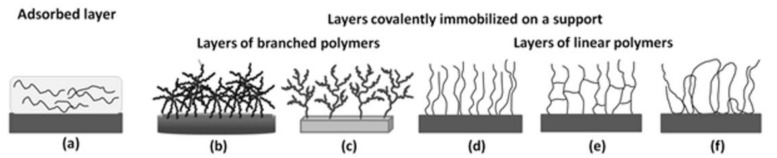
Different kinds of polymer layers immobilized on a support, obtained in the Laboratory of Nano- and Microstructural Materials: (**a**) adsorbed layer, (**b**) layer of star polymer, (**c**) layer of hyperbranched polymer, (**d**) layer of brush structure, (**e**) layers with crosslinked chains, (**f**) layer with interpenetrating polymer chains.

Polymer layers immobilized on a support consisted of polymers with branched or linear structures. They were physically and chemically attached to the surface (Figure 8).

The polymer layer can be easily created by casting the polymer solution on the solid substrate followed by evaporation of the solvent. Such a physically adsorbed polymer layer binds to the support, e.g., by weak electrostatic or van der Waals interactions. In our laboratory, we studied the stability of thermoresponsive polymer layers adsorbed on silica (Figure 8a) for their application in cell culture in vitro [83]. The polymer layer under cell culture conditions should be dehydrated, stable and undissolved, while after cell proliferation, the polymer should dissolve and be washed out. Considering our previous studies on the structure–property relationship of (co)poly(2-oxazoline)s [84], gradient copolymers of 2-isopropyl- with 2-n-propyl-2-oxazoline with T_CP_ below 37 °C in culture medium and a glass transition (T_g_) significantly above this temperature were chosen. The layers exhibited decreased wettability above the T_CP_, but the unexpected increase in the layer thickness indicated its gradient hydration: the part of the layer at the interface with the solid support was poorly solvated, while the layer in the outermost region was hydrated. Although the fibroblast adhesion and proliferation rate on partly hydrated (co)poly(2-oxazoline) films decreased compared with control layers, it was possible to separate cells from the matrices simply by lowering the culture temperature and dissolving the polymer. These studies highlighted the aspect of the stability of the polymer layer upon hydration, which is strictly related to the nature of its immobilization with a solid support.

We have studied different layers covalently bound with the support. For this purpose, first, the solid substrate was functionalized to introduce reactive species onto its surface. Then, by reacting these moieties with the monomer and further polymerization (“grafting from”) or with complementary groups in the previously synthesized polymer chain (“grafting to”), covalently bound layers were obtained. Layers with branched or linear (co)polymers were formed with thicknesses from a few nanometers to several hundred micrometers.

##### Layers of Linear Polymers

Linear polymethacrylates, poly(2-oxazoline)s and polyglycidol were immobilized on solid supports (silica, glass and polypropylene) using “grafting from” and “grafting to” methods.

##### The “Grafting from”—Layers of Brush Structures and Crosslinked Layers

**Figure 9:**
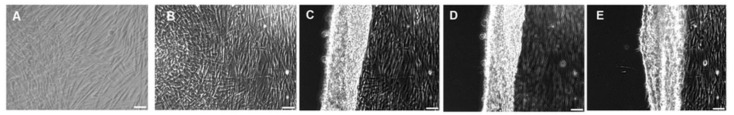
Images of fibroblasts on the (**A**) TCPS control layer, (**B**) P(TEGMA-EE) layer after 24 h of incubation at 37 °C and after (**C**) 5 min, (**D**) 10 min and (**E**) 20 min of incubation at 17.5 °C. The scale bars are 100 μm.

Two approaches of the “grafting from” method were applied to obtain the linear poly[tri(ethylene glycol) monoethyl ether methacrylate] (P(TEGMA-EE)) layers [85,86,87,88,89]. In the first case, initiator-functionalized glass and silica wafers were prepared, and α-bromoisobutyrate groups introduced on the surface were able to initiate the atom transfer radical polymerization of TEGMA-EE [86,87,88]. Layers of several nanometers of a polymer brush structure were obtained (Figure 8d). In the second case, polypropylene supports were treated with electron beam radiation to generate reactive species that initiated polymerization of TEGMA-EE and TEGMA-EE with the peptide sequence isoleucine–lysine–valine–alanine–valine (IKVAV) [85,89]. Crosslinked layers of several micrometers were formed (Figure 8e). All layers exhibited thermoresponsive properties: they were hydrated and swollen at 20 °C (below T_CP_) and dehydrated and shrunk at 37 °C (above T_CP_). Such thermoresponsive polymer layers were used for the culture of human fibroblasts [85,86,87] and amniotic stem cells [89] and for the coculture of fibroblasts with keratinocytes [88] and their detachment. Under culture conditions, cells adhered to and spread onto the P(TEGMA-EE) layers, forming a cell monolayer comparable to standard tissue culture polystyrene (TCPS) dishes (Figure 9A,B). Lowering the temperature below the T_CP_ of the immobilized polymer led to detachment of the cell sheet from the layer within several minutes (Figure 9C–E). The shortest fibroblast detachment time (10 min) was reached by P(TEGMA-EE) layers grafted on polypropylene [85]. Amniotic cells revealed a better tendency for full sheet proliferation on P(TEGMA-EE) layers and detachment than fibroblasts. The use of P(TEGMA-EE) with a peptide sequence IKVAV layer induced better cell confluence than the use of P(TEGMA-EE) itself, but worse detachment was observed. Genotoxicity assays indicated that no significant DNA damage of cells cultured and detached from P(TEGMA-EE) layers took place [87]. The studied procedure is a good alternative for enzyme detachment or scratching.

Fibroblast sheet detachment was accompanied by rolling of the sheet [87]. To prevent this and to support fibroblast transfer to a desired place, we proposed the use of a “transfer membrane”. After the cell culture was completed, the membrane was placed on a confluent cell sheet which caused the cells to adhere to it. Lowering the temperature caused detachment of the cells from the P(TEGMA-EE) layer and allowed for their transfer. This procedure did not influence cell viability. All these studies, performed in our laboratory, indicated that P(TEGMA-EE) layers immobilized on a support may successfully be used for engineering skin tissue, especially for delivering cell sheets in the treatment of burns and slow-healing wounds.

The “grafting to”—layers of interpenetrating polymer chains and layers of brush structure.

Linear (co)polymers of glycidol, poly(2-oxazoline)s and poly(ethylene glycol) with peptide were covalently immobilized on a solid support using “grafting to”. Two types of layers with polymer chains differently bound to the support were prepared.

The first type concerns interpenetrating polymer chains multiply attached to the support (Figure 8f). This type of layer was obtained from the linear homo- and copolymer of glycidol and its thermoresponsive derivative mPGL [90,91]. (Co)polymers were covalently attached by the esterification reaction between hydroxyl groups of (co)polyglycidols and anhydride groups introduced onto the surface. As -OH groups are distributed along the linear (co)polyglycidol chain, thus tethering to the support occurs at many points on the polymer chain. Layers of thickness in the range of 7–140 nm (dependent on polymer molar mass, concentration) were created [90]. Hydrophilic PGL layers were applied as antifouling materials against fibrinogen. Fibrinogen adsorption on surfaces was reduced by approximately 45–90% compared with the bare silica supports. Higher efficiency in the reduction of fibrinogen adsorption was found for surfaces covered with higher molar mass PGL. It was also interesting to further evaluate the possibility of controlling the affinity of the obtained polyglycidol-based layers to water and to “switch” this affinity depending on the external conditions. For this reason, thermoresponsive derivatives of polyglycidol mPGL were obtained [92,93,94,95] and then immobilized on a support [91]. Similar to the case of nonmodified PGL [90], tethering of the copolymer led to layers of interpenetrating polymer chains multiply attached to the support. The layer thickness and morphology could be easily controlled by the conditions applied during the preparation of layers. Below the T_CP_, layers were hydrated and swollen. The subsequent increase in temperature above the T_CP_ caused layer dehydration but, surprisingly, the layer thickness remained constant or was even slightly increased. It seemed that the removal of water from the interior of the layer was accompanied by the formation of hydrophobic interactions between polymer chains, which caused them to stretch instead of shrinking. This was probably due to the specific kind of polymer chain tethering with the support when compared to the layers of the brush structure. Under culture conditions (above T_CP_), fibroblasts adhered to and spread onto the dehydrated mPGL layers, forming a confluent cell sheet, while below T_CP_ the hydrophilic nature of layer did not favor the interactions with cells. 

The second type of linear polymer-based layer formed by “grafting to” concerns brush structures, where only one attachment point between the polymer chain and the solid substrate occurs (Figure 8d). The brush structure layers were obtained via termination of living chains by the functional groups present on the support or by the reaction between one reactive group at the polymer chain end and complementary functionality at the surface.

By the termination approach, brush layers of PGL and poly(glycidol-co-ethylene glycol) (P(GL-EO)) [90], as well as PIPOx [96,97] and poly[(2-ethyl-2-oxazoline)-co-(2-nonyl-2-oxazoline)]s (P(EtOx-NonOx)) [96], were prepared.

In the case of linear PGL and P(GL-EO), the living (co)polymer chains, with protected glycidol, were terminated by the chloropropyl groups anchored on the substrate. After deprotection, polymer layers with a thickness ranging from 1.1 to 2.3 nm were obtained [90]. Such nanolayers appeared to be a good candidate for antifouling materials, as decreased fibrinogen adsorption was observed compared to bare silica surfaces. The best antifouling properties were observed for densely grafted P(GL-EO).

The brush layers of poly(2-oxazoline)s (POx) were prepared by cationic ring opening homopolymerization of 2-isopropyl-2-oxazoline and copolymerization of 2-ethyl- with 2-nonyl-2-oxazoline, followed by termination of living chains by amino groups anchored on the surface [96,97]. Thermoresponsive, several nanometer-thick layers of semicrystalline morphology were obtained. It is known that poly(2-oxazoline)s are prone to crystallize both in bulk and in aqueous solutions, and this property is strengthened with increasing length of the 2-oxazoline linear side chain. Recently, crystallization of poly(2-ethyl-2-oxazoline) (PEtOx) was also described [98,99,100]. Among POx, PIPOx is especially prone to crystallize due to its chemical structure (the isopropyl substituent attached to the planar amide groups), which favors easy packing and, as a consequence, ordering of the polymer chains [101]. We have shown that PIPOx also exhibits the ability to crystallize in organic solvents, which occurred unexpectedly [102]. Our aim was to suppress the ability of PIPOx to crystallize both in the solid state and in solutions, as it potentially excludes this polymer from many applications. For that purpose, gradient copolymers of IPOx with 2-methyl-2-oxazoline, 2-n-propyl-2-oxazoline and ethyleneimine were obtained [103,104]. Although the copolymers exhibited a lower ability to crystallize compared to the homopolymer of 2-isopropyl-2-oxazoline, their T_CP_ changed significantly and, additionally, the prolonged incubation of the copolymers at an elevated temperature caused further crystallization. Recently, we showed that the random copolymerization of IPOx with 2,4-disubstituted-2-oxazoline significantly reduced the ability of the copolymer to crystallize compared to PIPOx while maintaining T_CP_ and T_g_ similar to those of PIPOx [105]. The copolymers were found to be nontoxic to fibroblasts at the concentrations required for biomedical tests. Such noncrystalline copolymers of 2-oxazolines exhibiting a phase transition around body temperature have not been obtained previously and could be interesting candidates for a wide range of biomedical applications. We noticed that the presence of crystalline fibril-like fibers on the PIPOx layers promoted the proliferation of fibroblasts more efficiently than noncrystalline P(EtOx-NonOx) [96]. Lowering the temperature of cell culture changed the morphology of fibroblasts, which was driven by the changes in the physiochemical properties of the PIPOx layer (shrinking and dehydration) and led to their detachment, similar to the case of P(TEGMA-EE) brush layers [86]. We observed that a high crystallite content, however, weakened the volume transition of the layer; thus, the effectiveness of detaching the cells from such a layer decreased. The unrolled cell sheet was detached and transferred with the use of a “transfer membrane”.

The brush layers were also created by the reaction between one reactive group at the polymer chain end and complementary functionality at the surface. This was applied to obtain poly(ethylene glycol) (PEG) conjugated with peptide layers [106,107]. First, a condensation reaction of a carboxyl group placed on one end of short PEG (DP up to 27) and the amines on the support was performed. The nonattached PEG chain end containing an amino group reacted with a fluorescently labeled pentapeptide of the glycine–arginine–methionine–leucine–glycine sequence. The length of the PEG chain significantly influenced the immobilization density of the peptide on the surface. Digestion of the peptide layers with trypsin led to the release of the fluorescent peptide fragment, which allowed for enzyme detection. The presence of the PEG linker between the peptide and the surface facilitated hydrolysis compared to peptides directly attached to the surface.

##### Nanolayers of Branched Polymers

In recent years, the preparation of nanolayers of branched polymers has been an increasingly widely studied issue. The nanolayers we studied were covalently bound to the substrate and remained stable under biological test conditions. The aim was to show how the chemical structure of the obtained hyperbranched and star polymers as well as their behavior on the surface, in some cases under the influence of solvent, pH and temperature changes, affect the possibility of using the obtained layers in gene therapy, antifouling and antibacterial applications.

Layers of star (co)polymers (Figure 8b) of OEGMA with DEGMA and glycidyl methacrylate (GMA) [65,72], DMAEMA [108] and hyperbranched polyglycidol (Figure 8c) [109] were prepared. Star (co)polymers were linked to the support by the “grafting to” method [65,72,108], while hyperbranched polyglycidol was linked by “grafting from” [109]. A schematic description of the properties of the obtained layers is provided in Table 3.

The layers made of poly(oligo(ethylene glycol) methacrylate) stars were thermoresponsive [65,72]. POEGMA star layers were used for culturing fibroblasts [65] or HT-1080 cells [72], and their noninvasive detachment was controlled only by changing the temperature [65,72]. The detachment of the fibroblast sheet from star POEGMA layers occurred faster than that from linear P(TEGMA-EE) brushes [86]. Thermoresponsive star POEGMA layers were also used for the deposition of DNA polymer carriers, which opened a route to the efficient delivery of nucleic acids into cells [72]. At an environmental temperature above the T_CP_ of the nanolayer and simultaneously the DNA polymer carrier, polyplex deposition was performed. The culture and transfection of HT-1080 cells was then carried out. It was observed that the nucleic acid was delivered into the cells and that the transfection efficiency was several times higher than that of the control. Moreover, transfected cells could be noninvasively detached using only environmental temperature changes.

PDMAEMA stars with different molar masses were grafted photochemically to solid supports and evaluated for antibacterial activity against Gram (+) Bacillus subtilis (Table 3) [108]. Linear PDMAEMA with various molar masses was grafted for comparison. Next, to enhance bactericidal properties, amino groups in both kinds of polymer layers were quaternized with bromoethane. The obtained PDMAEMA star nanolayers exhibited higher antimicrobial activity against Bacillus subtilis than the linear analogs [108].

The antifouling properties were investigated for hyperbranched polyglycidol grafted from silica and PET surfaces. The ring-opening polymerization of glycidol was induced by ionized hydroxyl groups attached to the surface. Additionally, the hydroxyl groups of immobilized hyperbranched polyglycidol were modified with hydrophilic poly(ethylene glycol) chains or hydrophobic ethyl carbamate groups to study the influence of such modification on protein adsorption. All hyperbranched polyglycidol-based layers were able to reduce fibrinogen adsorption by approximately 45–90% compared with bare supports [109]. The effectiveness in resisting protein adsorption depended on the grafting density, the type of modification of the peripheral polyglycidol hydroxyl groups and on the base materials onto which the polymer was grafted. The highest ability to reduce adsorption was observed for surfaces with densely grafted unmodified polyglycidol.

#### 2.2.2. Self-Supporting Layers

**Figure 10:**
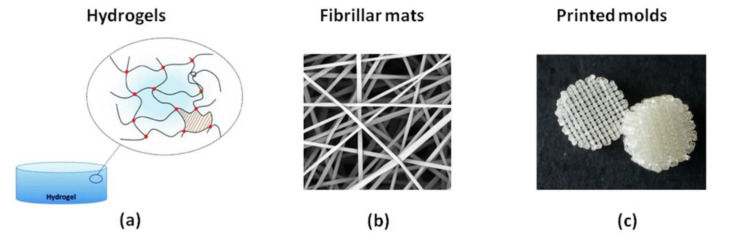
Different kinds of self-supported polymer materials obtained in the Laboratory of Nano- and Microstructural Materials: (**a**) hydrogels, (**b**) fibrillar mats, (**c**) printed molds.

In addition to layers immobilized on supports, self-supported polymer materials were also prepared in the Laboratory of Nano- and Microstructural Materials. Hydrogels, nonwoven fibrous mats and three-dimensional molds deserve our attention (Figure 10).

##### Hydrogels

Well-defined linear copolymers of glycidol were subjected to chemical crosslinking and photocrosslinking to obtain hydrogels. The very first study showed that the high degree of OH functionality in triblock copolymers of glycidol and ethylene oxide makes their crosslinking with glutar aldehyde possible [35]. By applying the sol–gel reaction of tetraethoxysilane (TEOS) within the swollen networks, a small amount of silica was incorporated in the hydrogels [110]. The addition of TEOS into hydrogels improved the gelation process and mechanical properties of the hydrogels. The hydrogels’ swelling capacity reached 400–3000%. The swelling ratio increased with an increase in the length of the PEO central block and with a decrease in the length of the PGL chains. It was suggested that sorption occurs mainly in the uncrosslinked PEO domains, but the hydroxyl groups remaining in the polyglycidol blocks after crosslinking also contribute to the swelling capacity [35,110]. The hydrogels were thermoresponsive, but a linear dependence of gel swelling/deswelling on temperature change was observed.

In our later research, the chemical crosslinking of the homopolymer of glycidol and its hydrophobically modified derivatives, which also exhibit thermoresponsiveness, was carried out using poly(ethylene glycol)-bis-(carboxymethyl ether chloride) as a crosslinking agent [111]. The equilibrium swelling degrees in water were much higher than those of hydrogels crosslinked with glutaraldehyde, which was attributed to long spacers of the poly(ethylene glycol) present in the network. Loosely crosslinked hydrogels of thermoresponsive copolymers displayed the most pronounced volume shrinkage with increasing temperature. All studied hydrogels [110,111] maintained their stability during several swelling/deswelling cycles, which makes them interesting for potential application.

Photocrosslinking was used to prepare hydrogels from homopolymers of glycidol and its thermoresponsive derivatives [112,113]. For that purpose, polymer mixtures with benzophenone (BP) or with its derivative (4-benzoylbenzyl) trimethylammonium chloride (BBTMAC), used as photosensitizers, were subjected to UV irradiation [113]. Upon absorption of a photon, BP or BBTMAC undergo photodissociation into radicals able to generate radicals on a polymer chain. The recombination of radicals and subsequent production of carbon–carbon bonds between the polymer chains cause crosslinking. With the cooperation of the Institute of Polymers of the Bulgarian Academy of Sciences, the photocrosslinking process was also carried out for a water polymer/photosensitizer mixture after freezing; thus, so-called cryogels were formed [112]. During freezing, water forms ice crystals, whereas soluble substances accumulate in a nonfrozen liquid microphase. Crosslinking occurs only in this microphase, and the ice crystals act as porogens. Such a procedure allowed us to minimize the exposure time and limited the possible degradation of the polymer compared to photocrosslinking without freezing [112,113]. Cryogels exhibited higher mechanical strength and a much faster reaction (seconds) to hydration and dehydration under temperature changes [113]. The cryogels exhibited satisfying interactions with cells and could be used as scaffolds for cell attachment and growth.

##### Fibrillar Mats

Another type of self-supported material with temperature-dependent behavior—polymer matrices in the form of nonwoven fibrous mats and three-dimensional molds—was prepared [83,114]. We aimed to obtain self-supported materials that are dehydrated, stable (preserving defined size, shape and porosity of the construct/scaffold/material) and undissolved under cell culture conditions. Additionally, after cell proliferation, when the temperature is decreased below the phase transition, the material could be simply washed out from the culture environment. Matrices in the form of mats were obtained by electrospinning and in the form of molds by fused deposition modeling (FDM). To obtain these materials, PIPOx and gradient copolymers of 2-isopropyl- with 2-n-propyl-2-oxazoline, with relatively low molar masses and low dispersity values, were processed. It was found [114] that the molar mass of all used (co)poly(2-oxazoline)s did not change after processing; however, prolonged extrusion by FDM at high temperatures caused polymer degradation. The thermal properties of all (co)poly(2-oxazoline)s changed significantly after electrospinning, and the content of the crystalline phase increased. All of the matrices remained undissolved when incubated in water at a temperature above the T_CP_, but an undesired loss of shape stability was observed. The fibrous mats were rolled up while the regular shape of the molds was distorted, and they collapsed into shapeless clods. Conversely, such significant distortions of the shape were not observed in the case of matrices in the form of layers adsorbed on silica slides [83] (see Section 2.2.1).

## 3. Summary

The works discussed in this article were conducted for many years in the Laboratory of Nano- and Microstructural Materials at the CMPW PAS under the supervision of Professor Andrzej Dworak. These studies have shown how the process of understanding the mechanisms of polymerization of oxiranes, cyclic imines and methacrylates through the synthesis of polymers with a defined structures and thus properties to obtaining nano- and microstructural materials could be carried out. In the case of substrates coated with thermoresponsive polymers for cell culture, the work has reached a high level of advancement, and cell culture technologies, designing tools for the transfer of cell sheets and preliminary documentation of the necessary approvals and certificates required for the final product have been prepared. The acquired knowledge and understanding of the behavior of the obtained materials in solution and on surfaces allowed us to propose the possibilities of their potential use in biology, diagnostics or regenerative and reconstructive medicine.

Due to the efforts and activity of Professor Dworak, the laboratory has developed cooperation with many recognized research centers, which is still ongoing. The works carried out in the Laboratory of Nano- and Microstructural Materials under the guidance of Professor Dworak put it at the forefront of the research groups involved in the study of polymer materials.

Studies on controlled polymerizations of oxiranes, cyclic imines and methacrylates in the Laboratory of Nano- and Microstructural Materials at the CMPW PAS have reached a high level of advancement and are still very intensive. These investigations will stimulate the future development of polymers beneficial for new applications.

We will continue Prof. Dworak’s legacy in our research on controlled synthesis of polymers and extend them to new linear and branched macromolecules. Their perspective applications, mainly in biomedical areas, is now an important part of our studies.

## Figures and Tables

**Table 1:** Polymer characteristics, experimental conditions and properties of mesoglobules prepared in the Laboratory of Nano- and Microstructural Materials of CMPW PAS.

Polymer Abbreviation	Polymer Specification	M_n_ (g/mol)	C_P_ (g/L)	Additive * s/p	R_h_ (nm)	Heating Mode	Ref.
POEGMA	P(T-ran-O_475_)_4.5	48,600	0.1	-	430	gradual	[16]
			0.5	-	875		
			0.1	-	95	abrupt (54 °C)	
	P(T-ran-O_475_)_30.5	46,800	0.1	-	800	gradual	
			0.5	-	1185		
			0.1	-	101	abrupt (81 °C)	
	P(D-ran-O_300_)_37	32,000	0.1	-	465	gradual	
			0.5	-	1080		
			0.1	-	96	abrupt (81 °C)	
P(HEMA-OEGMA)	-	33,000	0.5	-	740	gradual	[29,30]
				NaCl	1425		
				PBS	1540		
				-	230	abrupt (70 °C)	
				NaCl	1600		
				PBS	1330		
PETEGA	-	7000–40,000	0.5	-	177	abrupt (40 °C)	[21,31]
				-	91	abrupt (70 °C)	
			0.5	0.1	110	abrupt (40 °C)	
				0.1	70	abrupt (70 °C)	
			0.5	0.4	n.d	abrupt (40 °C)	
				0.4	10/71	abrupt (70 °C)	
-	P(D-co-A_A) 7% A	42,000	0.2	-	62	gradual	[25,26]
			0.5	-	77		
	P(D-co-A_Pr) 7% Pr	42,000	0.1	-	330	gradual	
			0.5	-	620		
			0.1	-	80	abrupt	
	P(D-co-A_Az) 7% Az	42,000	0.1	-	130	gradual	
			0.5	-	215		
			0.1	-	20	abrupt	
PIPOx	-	3660	0.5	0.5	403	abrupt	[22]
				-	494	abrupt (80 °C)	
				-	700	gradual	
		5540	0.5	0.5	370	abrupt (70 °C)	
				-	650	abrupt	
				-	800	gradual	
		8940	0.5	0.5	180	abrupt (70 °C)	
				-	800		
				-	1400	gradual	
mPGL	P(G-co-EGC)-28	800,000	0.05		175	abrupt (80 °C)	[17]
				0.2	260		
					175	dropwise (80 °C)	
	P(G-co-EGC)-35		0.05		112	abrupt (80 °C)	
				0.2	100		
					100	dropwise (80 °C)	
PNIPAM		84,000	1	0.05	45	abrupt (70 °C)	[20]
				0.5	10		

* Generally SDS unless otherwise stated.

**Table 2:** Star (co)polymers obtained in the Laboratory of Nano- and Microstructural Materials.

Core	Number of Arms	Arms	Properties/Application	Ref.
poly[p-(chloromethyl)styrene]	12–26	PEG	amphiphilic	[52]
poly[p-(iodomethyl)styrene]	10	PS, PtBuAc, PAA	PtBuAc stars: thermal properties PAA stars: amphiphilic/formation of reversible complexes between COOH groups of stars and model drug: cisplatin neurotoxicity evaluation electrical and rheological properties of star solutions	[38,53,54,55,66,67,68]
polyglycidol and dipentaerythritol	6, 13	PEOx	thermoresponsive	[46,64]
pentaerythritol derivatives	4, 6	tert-butyl glycidyl ether, glycidol	amphiphilic, thermoresponsive pyrene encapsulation formation of reversible complexes between OH groups of stars and Ru(NH_3_)_3_Cl_3_	[47,48,49]
aliphatic alcohols and calix[4]arenes	3, 4, 6, 12, 16	PtBuAc	branching parameters, scaling equations	[50,51]
PArOx	20, 22	P(DEGMA-*co*-OEGMA)	amphiphilic, thermoresponsive, nontoxic/encapsulation of fluorescent probe	[56,65]
PArOx	28	PtBuAc, PtBuMAc, PAA and PMA	polyacid stars: pH responsive	[61,62,63]
PArOx	28	PDMAEMA	thermo- and pH responsive, nontoxic/gene delivery	[57,59]
PArOx	28	PDMAEMA with AgNPs	antibacterial agents	[71]
PArOx	28	P(DMAEMA-*co*-DEGMA) P(DMAEMA-*co*-OEGMA-OH)	thermo- and pH responsive, nontoxic, gene delivery	[58,59,60,70,72]

**Table 3:** Characteristics of the linear and branched polymer layers immobilized on a support.

Polymer	M_n_ [g/mol]	Support	Type of the Layer	Thickness of the Layer	Grafting Density	Application	Ref.
**grafting to**							
PGL	1,900,000	silica	interpenetrating polymer chains	15–120 nm	-	reduce fibrinogen adsorption	[90]
PGL	8000	silica	interpenetrating polymer chains	7–140 nm	-	reduce fibrinogen adsorption	[90]
PGL	8000	silica	brushes	1.1–1.5 nm (+/−0.3 nm)	0.083–0.113 chains/nm^2^	reduce fibrinogen adsorption	[90]
P(Gl-EO)	6000	silica	brushes	1.7–2.3 nm (+/−0.3 nm)	0.17–0.23 chains/nm^2^	reduce fibrinogen adsorption	[90]
mPGL	2,200,000	silica, glass	interpenetrating polymer chains	20–60 nm	-	the growth of fibroblasts and keratinocytes	[91]
P(EtOx-NonOx)	14,000–21,800	glass	brushes	4–8 nm	0.19–0.22 chains/nm^2^	the growth and harvesting of fibroblasts	[96]
PIPOx	20,800–42,000	silica, glass	brushes	5–11 nm	0.16–0.26 chains/nm^2^	the growth and harvesting of fibroblasts	[96,97]
PEG	576–1545	silica	brushes	-	2.88–3.54 pmol/mm^2^	proteolytic enzyme detection	[106,107]
P(DEGMA-*ran*-OEGMA-*ran*-GMA)	380,000	silica, glass	star polymer	58 nm	-	the growth and harvesting of fibroblasts	[65]
P(DEGMA-OEGMA)	417,000	silica	star polymer	30 nm	-	deposition gene delivery and the growth and harvesting of HT-1080	[72]
PDMAEMA	9000–1,000,000	silica, glass	linear and star polymer	3–100 nm	-	antibacterial	[108]
**grafting from**							
P(TEGMA-EE)	23,000–189,000	silica, glass	brushes	3–18 nm	0.1 chain/nm^2^	the growth and harvesting of fibroblasts and coculture of fibroblasts and keratinocytes	[86,87,88]
P(TEGMA-EE)	-	polypropylene	crosslinked layer	>10 μm	1.15 +/−0.03 mg/cm^2^	the growth and harvesting of fibroblasts and amniotic stem cells	[85,89]
PGL	1600–290,000	silica, PET	hyperbranched	4–5 nm		reduce fibrinogen adsorption	[109]

## Data Availability

Data available on request.
